# High-efficiency antibody discovery achieved with multiplexed microscopy

**DOI:** 10.1093/jmicro/dfw014

**Published:** 2016-09-01

**Authors:** Shelley Mettler Izquierdo, Stephanie Varela, Minha Park, Ellen J. Collarini, Daniel Lu, Shreya Pramanick, Joseph Rucker, Lucia Lopalco, Rob Etches, William Harriman

**Affiliations:** 1 Crystal Bioscience, Emeryville, CA, USA; 2 Integral Molecular, Philadelphia, PA, USA; 3 Division of Immunology, Transplantation and Infectious Diseases, San Raffaele Scientific Institute, Milan, Italy

**Keywords:** antibody discovery, single B cell cloning, chicken monoclonal antibody, ion channel, GPCR, DR4 DR5

## Abstract

The analysis of secreted antibody from large and diverse populations of B cells in parallel at the clonal level can reveal desirable antibodies for diagnostic or therapeutic applications. By immobilizing B cells in microdroplets with particulate reporters, decoding and isolating them in a microscopy environment, we have recovered panels of antibodies with rare attributes to therapeutically relevant targets. The ability to screen up to 100 million cells in a single experiment can be fully leveraged by accessing primary B-cell populations from evolutionarily divergent species such as chickens.

## Introduction

The ‘age of monoclonal antibodies’ was ushered in with the advent of hybridoma technology in the 1970s. In the following decades, a myriad of monoclonal antibodies (mAbs) have been discovered and applied as reagents, diagnostics and therapeutics. The therapeutic market exceeded US$ 75 billion in 2014, and there are more than 300 antibody products in development. The compounded annual growth rate of the mAb market is 8% [1]. Throughout this period of rapid growth, antibody discovery has relied heavily on classical hybridoma technology in spite of three significant limitations. The first is the potential four-log loss of repertoire during the cellular fusion process that vastly reduces the likelihood of finding mAbs with rare attributes. Second, targets that are highly conserved between mammals are often poorly immunogenic in rodents. Finally, it is generally difficult using hybridoma technology to raise antibodies that are rodent cross-reactive, a necessary property for testing in many animal models of disease.

To address these limitations, numerous alternative antibody discovery approaches have been developed, most notably phage and ribosome display [2–4] and more recently yeast [5,6] and mammalian cell display [7–9]. Antibody libraries have been made with either natural or synthetic antibody sequences [10–12], including human-derived sequences [13,14]. While some naive libraries can be very large in size, with sizes estimated at over 10^11^ members, antibodies discovered from these sources often require additional *in vitro* affinity maturation to reach potency comparable to traditional hybridoma antibodies [15]. In some cases, phage libraries have been made with affinity-matured sequences from immunized animals [16–20]. However, the original heavy and light chain pairings found in the B cells are generally lost in the cloning process, resulting in diminished diversity of antigen-specific clones and, in the absence of *in vivo* affinity maturation, an increased frequency of off-target reactivity [21,22].

For the above reasons, we developed a technology capable of interrogating the entire population of splenic B cells from immunized animals. The platform was designed to (i) screen up to 100 million cells in a single experiment; (ii) recover antibody-secreting B cells directly from tissue of any species without immortalization, transformation, stimulation or extended cell culture; (iii) facilitate multiparameter screening of antibody characteristics from a single B cell; (iv) directly identify biologically active antibodies; (v) screen for antibodies binding with low nanomolar affinity or better; (vi) retain the heavy and light chain pairs created by *in vivo* affinity maturation and (vii) select 100 or more clones within a few hours after harvesting B cells from an immunized animal. We refer to the platform as the gel encapsulated microenvironment (GEM) assay and herein describe its application to several antigen targets, demonstrating its versatility and power to find rare antibodies to unique epitopes.

We have used the GEM assay successfully on chicken, human, llama, rat and mouse B cells. The projects described here are all from chicken immunizations. Chickens have long been recognized as an excellent option for targets that are highly conserved in mammals [23–33]. However, chicken antibodies have historically been polyclonal preparations because classical hybridoma technology has not proven to be robust in the chicken system [34]. With the advent of single-cell technologies such as GEM delivering chicken mAbs, it has been shown that epitope recognition of human targets is often enhanced in immunized chickens over mammals, because the evolutionary distance allows them to recognize epitopes that are common to mammals [35]. In addition, immunized chickens are advantageous because they routinely produce antibodies with affinities in the low nanomolar to picomolar range [35].

## Methods

### Immunization of chickens

For each target antigen, two female White Leghorn chickens (8–9 weeks of age) were dosed every 2 weeks with the specified antigen for a total of six injections per animal. Protein antigens (ACRO Biosystems) were boosted intramuscularly (IM) at 200 µg per injection (with Freund's complete adjuvant for the initial boost and incomplete adjuvant for subsequent boosts). For DNA immunization, 1 µg plasmid DNA encoding full-length human CCR5 cloned into the expression vector pcDNA3.1 was coated onto 1 µm gold particles and administered into the dermis slightly dorsal to the ventral feather tract using the Helios Gene Gun System (Bio-Rad) following the manufacturer's protocol. Viral lipoparticles (VLPs, Integral Molecular, Inc.) were dosed IM at 300 µg for the initial boost and 150 µg for the remaining boosts. Plasma samples were collected bi-weekly to determine the titer in ELISA format. All immunization protocols have been approved by the Crystal Bioscience IACUC. Chickens were monitored routinely and were absent of signs of discomfort and illness throughout the course of immunization.

### Screening single antibody-secreting B cells using the GEM assay

#### Preparation of reporter beads

Five-micrometer polystyrene beads (Life Technologies) were coated with purified protein at a 5× mass ratio. VLPs were coated onto beads at 25 units per 40 µg beads. After coating, beads were washed and blocked with sterile-filtered 3% milk powder in PBS.

#### Staining reporter cells with vital dye

For either CHO cells or Jurkat cells, 2–5 × 10^7^ cells were washed with 10 ml of PBS and resuspended in 35 ml of serum-free RPMI-1640 (Mediatech) containing 20 µM Cell Tracker blue (Life Technologies) and were incubated at 37°C for 40 min. The cells were washed with 5 ml of PBS followed by 5 ml of trypsin (Mediatech). The dyed cells were resuspended in 60 ml RPMI growth medium, consisting of RPMI-1640 medium (Mediatech) supplemented with 10% fetal bovine serum (FBS; Hyclone), 1× Pen/Strep, 1× Glutamax, 1× NEAA (all from Life Technologies), and incubated overnight at 37°C for use in GEMs the following day.

#### Preparation of splenocytes

The capsule of the spleen was removed in cold 1% BSA/PBS and cut into four to five pieces with scissors. The pieces were rubbed over a 70-µm mesh to make a single-cell suspension in 1% BSA/PBS. The cells were centrifuged at 400 × *g* for 5 min and resuspended in 24 ml of PBS. Twelve milliliters of the suspension were overlayed onto 12 ml of histopaque 1077 (Sigma cat # 10771-500 ml) in two 50 ml conical tubes. The preparation was centrifuged at 400 × *g* for 30 min. Lymphocytes were collected from the interface, resuspended in 20 ml of 1% BSA/ PBS, and counted. Cells not used within a few hours were frozen in aliquots of 2 × 10^8^ spenocytes/ml in 10% DMSO diluted in RPMI growth medium. Freshly thawed cells from cryopreserved aliquots perform equivalently to freshly isolated spleen cells in both GEM and subsequent molecular biology steps for a period of up to 24 h.

#### Preparation of GEMs

GEMs were prepared using a variation of the gel microdroplet process described previously [36–38] and US Patents 8,030,095 and 8,415,173. Ten to fifty million splenocytes were mixed with reporter beads (4 × 10^7^–3 × 10^8^) or reporter cells (1–2 × 10^7^). This suspension was mixed with a low melting point agarose at 37°C and added dropwise to an excess volume of dimethylpolysiloxane (Sigma) while mixing to generate an emulsion. Emulsified droplets were solidified by continued mixing on ice, and then the emulsion was overlaid onto RPMI growth medium and centrifuged at 400 × *g* at 4°C for 10 min. The oil phase was removed, and GEMs were washed 3× with 1% BSA/PBS and transferred to into complete RPMI growth medium. Goat anti-chicken IgY (Alexa 488 or Alexa 594; Life Technologies) was added to the GEM suspension at a dilution of 1:1000 and incubated at 37°C for 3–12 h, depending on the requirements of the particular assay. For incubations of this duration, no growth factors are needed for survival of the antibody-secreting cells.

#### Visualizing GEMs and recovering B cells of interest

The entire GEM prep was washed 3× in 1% BSA in PBS, transferred into manipulation media (CO_2_ independent medium, Life Technologies; 10% FBS (Hyclone), 1× Glutamax, 1× Pen/Strep; all from Life Technologies) and dispensed into a single 128 × 86 mm OmniTray (Nunc) tissue culture dish and scanned by eye at 50× magnification on a Leica DMI 6000 fluorescence microscope using a B/G/R triple filter (Chroma). GEMs containing profiles of potential interest were examined at 100× or 200× magnification to confirm profiles. GEMs featuring the desired reporter profile were harvested directly from the dish using a hand-held micropipettor and placed into a well of a 96-well plate containing 6 M guanidium thiocyanate lysis buffer, which rapidly dissolves the GEM matrix, lyses the secreting lymphocytes and preserves the cellular mRNA.

### Cloning, expression and initial characterization of recombinant mAbs

#### RT-PCR from single cells

mRNA from single cells was purified by oligo-dT capture and used to generate cDNA, which was then used as template for subsequent PCR reactions (OneStep RT-PCR kit, Qiagen). For each GEM isolated, the expressed chicken VH and VL genes were amplified and assembled with overlap PCR to generate a recombinant scFv antibody as previously described [17] and further assembled with a human Fc sequence to generate a scFv-Fc expression cassette which was cloned into the mammalian expression vector pF5a (Promega). Primer sequences are as follows: light chain forward (includes leader peptide): 5′ATGGCCTGGGCTCCTCTCCTC3′; light chain reverse (FW4 region): 5′TAGGACGGTCAGGGTTGTC3′; heavy chain forward (FW1 region): 5′ATGGCGGCCGTGACGTTGGA 3′ and heavy chain reverse (FW4 region): 5′GGAGGAGACGATGACTTCGGTCC 3′.

#### Antibody expression

Plasmids containing recombinant scFv-Fc from the GEM harvests were transiently transfected into HEK 293 cells grown in Freestyle F17 medium or Expi293 cells grown in Expi293 expression medium in 96-well format using 293fectin or Expifectamine (cells, media and transfection reagents, all from Life Technologies) following the manufacturer's instructions, and clonal supernatants were harvested. Supernatants were tested for antigen binding activity by ELISA and/or FACS. All clones that were confirmed to bind their respective targets were fully sequenced to avoid redundancies. Confirmed unique clones were re-transfected at a 2 ml volume to yield 200–500 µg recombinant antibody that could be purified as needed for downstream assays.

### Antibody characterization

#### DR4/DR5

*Cross-reactivity*: ELISA plates were coated with DR4, DR5 or OPG at 2 µg/ml in PBS, blocked with PBST/3% milk powder and detected with anti-huFc-HRP (Sigma).

*Apoptosis assay*: Jurkat cells (a human T cell line; ATCC) were grown in RPMI-1640 (Mediatech) supplemented with 10% heat-inactivated FBS (Life Technologies), 10 mM HEPES (Mediatech) and dispensed in a 96-well plate at 100K cells per well in the presence of test antibody at 10 µg/ml starting concentration (followed by serial dilutions) and incubated 4–12 h at 37°C. After incubation, CaspACE-FITC (Promega) was added to a final concentration of 5 µM for 20 min and washed before reading on a fluorescence plate reader.

#### CCR5

*Cell lines*: Cf2Th/synCCR5 cells, derived from the canine thymocyte cell line Cf2Th, were obtained from the NIH AIDS Research and Reference Reagent Program and were cultured in DMEM, 10% FBS, 1× Glutamate, 1× NEAA, 1× Pen/strep, 500 µg/ml G418, 500 µg/ml zeocin and 3µg/ml puromycin (Invitrogen). CCR5-transduced SupT1-M10 [39] and parental SupT1 (not transduced with CCR5) were kindly provided by H. Garg.

*Cell surface binding*: CCR5 expressing and non-expressing control cells were incubated with antibody supernatants for 1 h on ice, washed and incubated with rabbit anti-human Ig FITC (Sigma) for 1 h on ice, washed and analyzed by flow cytometry.

*Long-lasting internalization of CCR5*: Receptor internalization was induced by exposing SupT1-M10 cells to supernatants containing mAbs to CCR5 for 48 h at 37°C. To verify receptor downregulation, cells were also incubated with Mip1beta (CCR5 ligand) for 1 h. As positive control of long-lasting internalization, a previously described pool of mouse antisera to CCR5 was used [40].

#### P2X3

*Cell surface binding*: HEK cells expressing human or rat P2X3, or human P2X2, and control cells were incubated with antibody supernatants for 1 h on ice, washed and incubated with rabbit anti-human Ig FITC (Sigma) for 1 h on ice, washed and analyzed by flow cytometry.

*Inhibition of Ca^++^ signaling*: Antibodies were tested for ability to inhibit α,β-meATP-induced calcium flux using a cell-based Ca^2+^ flux assay [41]. Briefly, canine Cf2Th cells were transfected with the appropriate expression vector in poly-lysine coated, black 384-well plates with clear bottoms (Costar). Cells were incubated for 22 h at 37°C then washed twice and loaded with a calcium indicator dye in HBSS containing 20 mM HEPES (Calcium 4 Assay kit, Molecular Devices), incubated for 1.5 h, then moved to a Flexstation II-384 (Molecular Devices) set at 32°C. After a 10-min temperature equilibration, the specific P2X3 agonist α,β-meATP (Sigma) was injected (at *t* = 20 s) and fluorescence was measured for 60 s (reading every 3 s). Data sets were analyzed using Prism 5.0 software (GraphPad Software, Inc).

## Results

**Fig. 1.**
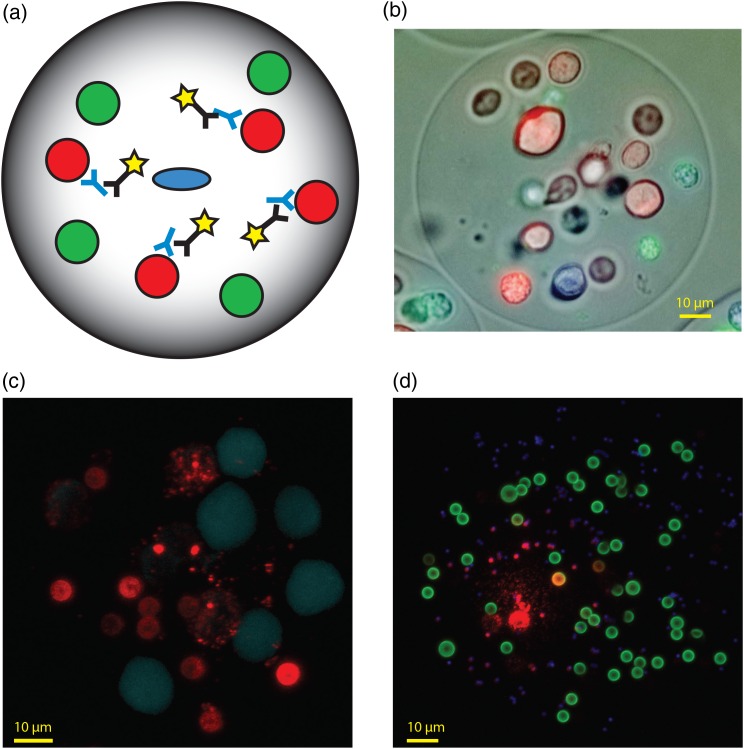
The GEM assay and selected variations. (a) Schematic representation of a GEM containing a single antibody-secreting cell (blue) and multiple reporter beads (red and green) coated with antigens of interest. Secreted antibody that binds to reporter beads is detected with anti-Ig secondary antibody (yellow). (b) Composite image of a GEM containing alternative reporter cells, dyed with either blue or red vital dye, and a green biological reporter (Caspase-3 FITC) that co-localizes with the blue cells, indicating this population is apoptotic. (c) Fluorescent image of a GEM containing 4.5 µm white beads coated with purified receptor, and non-dyed CHO cells expressing the same receptor, and blue-dyed parental CHO cells that do not express the receptor. Red secondary antibody can be detected on beads and non-dyed cells but not blue control cells. (d) Fluorescent image of a GEM containing reporter beads of three different sizes/colors (1.4 µm, blue; 4.5 µm, green; 9.0 µm, white). Co-localization of detection antibody (red) on alternative reporters produces distinctive hues.

The GEM assay consists of a single antibody-secreting B-lineage cell in a droplet containing multiple particulate reporters. The reporters can be beads, cells or other particles, which are immobilized within the matrix of the GEM. Antibody is secreted from the B cell and diffuses locally within the GEM where it binds to its target displayed on the reporter. Reporter-bound antibody is detected with a labeled secondary antibody (Fig. 1a). Typically, at least two types of reporters are used to confirm specificity. GEMs are made in batches accommodating as few as several thousand or as many as 100 million total cells, but experiments are generally performed with 10–50 million cells when using splenocytes. GEMs are viewed on the microscope, manually harvested and placed directly into a lysis buffer to preserve mRNA, in as little as 6 h of being in the live animal. Clonal derivation of the antibody is achieved by amplifying RNA encoding the chicken V regions of the heavy and light chains by RT-PCR and expressing them in a single-chain format with a human IgG1 constant region (scFv-Fc). This is the format we typically use, but other species/isotype combinations have been generated. It should be noted that the chicken's genetic architecture and its mechanism of diversification greatly facilitates V gene recovery from single cells. Since all the B cells in a chicken start with a single ‘primordial’ functional V gene at each of the heavy and light chain loci that is diversified through gene conversion using upstream pseudogenes, non-degenerate PCR primers can be designed solely based upon the sequence of the primordial VH and VL genes. This both reduces the complexity of the oligo cocktail and improves amplification efficiency.

### Example 1: antibodies to DR4 and DR5

#### Background

The TRAIL receptors R1 and R2, also known as DR4 and DR5, respectively, are members of the TNF receptor family [42]. Binding of the ligand TRAIL can induce apoptosis in a variety of cell types and agonistic antibodies to these receptors are also pro-apoptotic [43–45]. Other TRAIL receptors such as R3 are considered decoy receptors because they lack the intracellular signaling domain and antagonize TRAIL-induced apoptosis [46]. In addition, soluble forms of the TNFR superfamily, such as osteoprotegerin (OPG), can also act as decoy receptors [47].

#### Design goals

We aimed to find potent agonistic antibodies that could signal through both DR4 and DR5 but were not inhibited by decoy receptors of the TNFR superfamily. Because DR4 and DR5 share only 63% identity at the protein level, we anticipated that finding mAbs meeting these criteria would be challenging.

#### Immunization

Chickens were immunized with an alternating regimen of human DR4-Fc protein and human DR5-Fc protein at 2-week intervals. Spleens were taken from animals after 8 weeks, when titers reached >1:100,000 to both targets.

*GEM strategy*: GEMs were set up in a variety of ways (see below), but in all cases DR4 and DR5 were coated onto white or blue reporter beads, respectively. Clones were preferentially recovered when the secondary antibody (goat anti-chicken IgY Alexa 594) was seen on both bead types. Our primary strategy was to pick DR4/DR5+ clones, although we also selected DR4-only and DR5-only clones to confirm specificity of the GEM screen. To eliminate anti-Fc false positives, DR4 and DR5 proteins were coated on the beads as purified ECDs, not Fc fusions. In one variation of the GEM assay a third colored bead coated with OPG was used to avoid ‘pan-TNFR’ specificities. Another variation included Jurkat cells, which express DR4 and DR5. Cells that became apoptotic due to agonistic agents were detected with Caspase 3-FITC, showing green fluorescence within the GEM (Fig. 1b). In GEMs with cells and beads, we focused on DR4/DR5+ clones that also contained green apoptotic cells. In all cases, we avoided GEMs with a signal on the OPG beads. OPG cross-reactivity was seen in only ∼20% of the GEMs, so it was relatively easy to avoid. The desired cross-reactivity of DR4/5+ was rare (2–5% of single positive clones). GEMs showing apoptotic signal in the reporter cells were seen in <5% of the events where DR4 and/or DR5 reactivity were seen on beads.

#### Antibody profiles

**Fig. 2.**
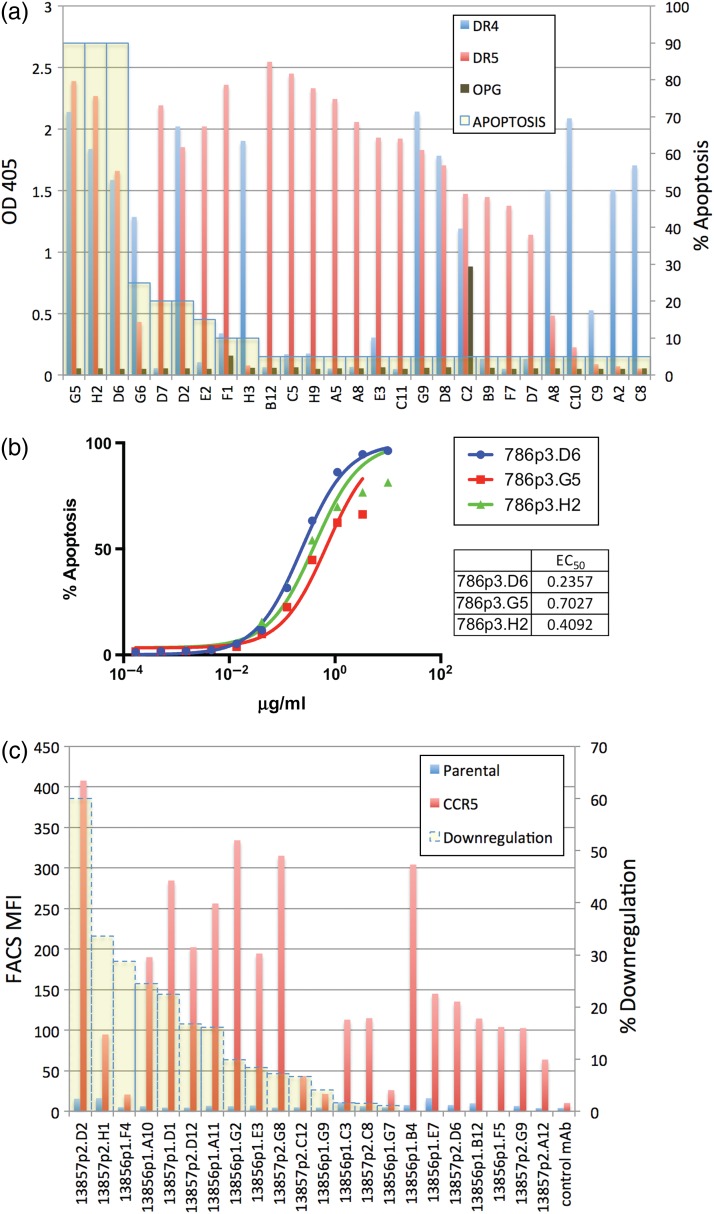
Specificity and activity of DR4/DR5 antibodies and CCR5 antibodies. (a) Left axis is ELISA OD values on DR4, DR5 and OPG coated plates; right axis is percentage of apoptotic Jurkat T cells after overnight incubation in supernatant of each clone. (b) Apoptosis assay dose response of three selected DR4/DR5 cross-reactive clonal supernatants on Jurkat T cells. (c) Specificity and activity of CCR5 antibody clones. The left axis is FACS staining SupT1 CCR5-M10 and parental cells; the right axis is percentage of downregulation of surface CCR5 expression on SupT1 CCR5-M10 cells after 48 h incubation.

Recombinant antibody supernatants were evaluated for binding in ELISA to DR4 and DR5, and all hits were sequenced. Twenty-seven unique sequence clones were re-transfected and confirmed in ELISA with DR4/DR5/OPG and binding profiles generated (Fig. 2a). These clones were also evaluated for pro-apoptotic activity in a single point assay at 10 µg/ml. In this cohort, we confirmed six clones to be DR4/5+, but only three showed significant pro-apoptotic activity. These three clones were chosen for EC_50_ analysis in the apoptosis assay (Fig. 2b), and they ranged between 236 and 703 µg/ml, showing improved potency relative to that of Lexatumumab, a clinical-grade DR5 antibody with an EC_50_ of 830 µg/ml on Jurkat cells [48].

The GEM assay identified cross-reactive antibodies to DR4 and DR5 but not pan-TFNR superfamily activity. Not only do these antibodies recognize a rare common epitope, some of them are also pro-apoptotic. The ability to obtain antibodies with this precise profile attests to the multiplexing capability of the GEM screen.

### Example 2: antibodies to CCR5

#### Background

Entry of human immunodeficiency virus type 1 (HIV-1) requires host cell expression of CD4 and a fusion co-receptor such as the G-protein-coupled receptor (GPCR) chemokine receptors CCR5 or CXCR4. CCR5 is the predominant co-receptor during the early stages of infection, and half or more of all infected individuals progress to AIDS harboring only CCR5 (R5)-tropic viruses. Epidemiological studies clearly demonstrate that CCR5 plays a critical role in HIV-1 transmission and pathogenesis *in vivo*. Individuals who possess two copies of a non-functional CCR5 allele (the Δ32 allele) are strongly protected against infection by HIV [49,50]. It has been shown that natural CCR5-specific Abs, found in HIV infection in human [51] or experimentally elicited in animal models [52,53], can induce a long-term downregulation of CCR5 surface expression, resulting in a ‘Δ32-like’ phenotype that can confer resistance to HIV infection [54].

#### Design goals

As a class, GPCRs are difficult to raise antibodies against due to their multipass transmembrane structure. Antibodies raised against peptides or single loops often do not recognize the full native structure. We aimed to develop a panel of antibodies capable of specifically binding native confirmation CCR5 on the cell surface as well as promoting receptor internalization.

*Immunization*: Native structure GPCR is difficult to purify in amounts sufficient for multiple boosts in animals, so DNA immunization was used. Titers reached a peak of ∼1: 5000 after five or six DNA boosts. Final boosts were performed with purified CCR5 protein presented on synthetic liposomes.

#### GEM strategy

Stably transfected Cf2Th/synCCR5 cells highly expressing CCR5 were used as the primary cellular reporter in the GEM assay, with the parental Cf2Th cell line as a negative control reporter to identify off-target antibodies. The two types of cells were differentiated by the use of a vital dye in the control cells (see example, of cellular reporters in Fig. 1c).

#### Results

Recombinant antibody supernatants were evaluated for binding by FACS to Cf2Th/synCCR5 cells, and all hits were sequenced. Thirty-six unique sequence mAbs were initially identified and then further evaluated by binding to other CCR5 expressing cell lines including SupT1 CCR5-M10 cells, to which 23 clones specifically bound (Fig. 2c). It has been demonstrated that anti-CCR5 Abs directed to the extracellular loop 1 domain can induce a long-lasting internalization of the receptor (48 h), whereas all known CCR5 modulating molecules show short-term kinetics (60–90 min) [54]. We evaluated the ability of these mAbs to induce long-lasting internalization of CCR5; five of these clones induced a >20% downregulation of the receptor after 48 h incubation and one of them a >60% downregulation. Of note, this latter mAb shows the highest level of binding on native CCR5 (Fig. 2c).

Despite the general difficulties in raising mAbs to GPCRs, a small campaign using the GEM assay with color-coded CCR5+ and CCR5− cells as reporters quickly yielded a substantial panel of antibodies. The use of DNA immunization obviated the need for large amounts of purified protein, although the titer was not as high as typically observed after conventional immunization. Nevertheless the rare antibody-producing cells generated by DNA immunization were recovered using the GEM assay.

### Example 3: antibodies to P2X3

#### Background

P2X3 is a ligand-gated ion channel that is important for peripheral pain responses [55]. P2X3 antagonists may be therapeutic for neuropathic pain, although to date small molecules have not demonstrated sufficient specificity to be effective. A highly specific and antagonistic P2X3 mAb therefore has potential therapeutic value.

#### Design goals

The desired antibodies should (i) bind human P2X3; (ii) act as antagonists to block signaling through the receptor and (iii) should not bind other members of the P2X family. In addition, cross-reactivity to P2X3 of other species, including rat and mouse, would facilitate evaluation of the antibodies in animal models of disease.

#### Immunization

Chickens were immunized with P2X3 VLPs (Integral Molecular, Inc.) that were produced from an overexpressing cell line. VLPs contained native conformation P2X3 as well as other naturally occurring membrane components. After four boosts with P2X3 VLPs, a serum titer of 1:12,500 was seen, representing a >5× signal on P2X3 VLPs as compared with control VLPs produced from cells that do not express P2X3.

#### GEM strategy

The serum ELISA from immunized animals showed significant reactivity to null VLPs, indicating that immune recognition of off-target antigen constituents had occurred. To avoid recovering mAbs with these off-target specificities, null VLPs were coated onto beads for a negative control. A different color bead coated with P2X3 VLPs was used as a positive indicator. Only GEMs showing antibodies binding to the P2X3 beads and not the null VLP beads were recovered for antibody gene amplification.

#### Results

**Fig. 3.**
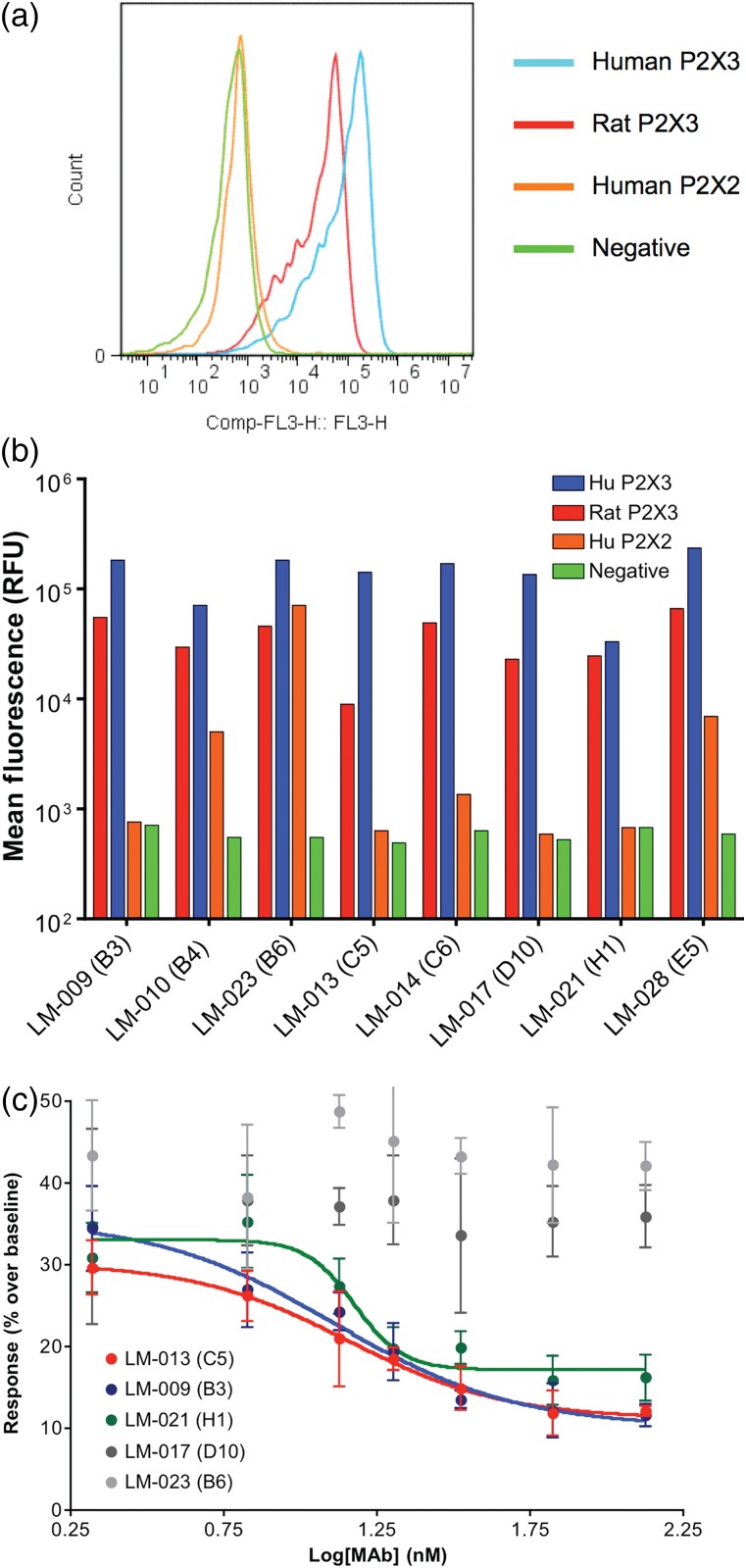
Specificity and activity of P2X3 antibodies. (a) Representative flow cytometry plot of test antibody (clone LM-009 shown) staining of HEK 293 cells expressing either human or rat P2X3, or human P2X2; non-expressing control cells are also shown. (b) Mean fluorescence shown for a panel of 8 mAbs evaluated in flow cytometry on P2X3, P2X2 or parental HEK 293 cells. All clones were cross-reactive for human and rat P2X3, with one clone also binding strongly to human P2X2. (c) P2X3 mAb showing dose-dependent inhibition of signaling (Ca^++^ response) through the receptor in transfected Cf2Th cells.

Over 25 unique sequence antibodies that showed a strong differential between P2X3 VLPs versus null VLPs by ELISA were recovered in the initial mAb screen. Eight clones representing the most diverse sequences in the panel were selected for further analysis. All eight clones labeled cell lines expressing human P2X3 as well as rat P2X3 (Fig. 3a and b). One clone showed strong cross-reactivity to human P2X2, and two others showed slight cross-reactivity. The remaining clones appeared specific for P2X3. In functional assays measuring calcium flux, four of the eight clones demonstrated a dose-dependent inhibition of P2X3-mediated signaling; three are shown in Fig. 3c.

In this case, the GEM assay was configured to find specific antibodies by having a reporter for off-target antigens as well as one for the target antigen. Not only did the strategy yield a significant number of P2X3 specific antibodies that were species cross-reactive, a substantial fraction of the clones exhibited biological activity as well.

## Discussion

For any therapeutic antibody discovery campaign, it is beneficial to have as wide a range of epitopes covered as possible, and having multiple antibody clones for each of those epitopes provides the highest likelihood of identifying a best-in-class antibody. In each case reported herein, the evolutionary distance between chickens and humans generated an exceptionally broad and diverse immune repertoire. We were able to sample this repertoire deeply in a microscopy environment by interrogating large numbers of primary B cells using multiple reporters in GEMs.

A chicken spleen contains ∼2–4 × 10^9^ lymphocytes. In the above examples, aliquots of 10^7^–10^8^ splenic lymphocytes were used within hours of collection with the remainder cryopreserved for future use. Around 0.3% of the cells in a GEM preparation are B lineage cells that secrete sufficient antibody to be detected in GEMs. Depending on the antigen and robustness of immunization, we may find fewer than 100 to more than 1000 antigen-specific GEMs in a given GEM preparation. As the desired antibody profile becomes better defined through a more complex set of reporters, the number of ‘worthy’ GEMs may be reduced to <10.

In the case studies presented here, we obtained a wide range of final serum titer (1:5000 for CCR5, 1:12,500 for P2X3 and >1:100,000 for DR4/5), which positively correlated with the frequency of antigen-specific GEMs seen for each target. The titer discrepancy is likely influenced by both the structure of protein (e.g. globular or multitransmembrane pass) and the type of antigen (purified protein, membrane-associated protein or DNA). DNA immunizations generally provide lower final titers and fewer GEMs than other immunogens, but this does not necessarily compromise the quality of antibodies recovered with respect to epitopic diversity or affinity. We routinely see antibodies to any of these target and immunogen types with *K*_d_s of 10 nM or better.

Although spleen cells are used for our chicken antibody projects, we have made GEMs using human, llama, rat and mouse peripheral blood lymphocytes or spleen cells. Additionally, murine hybridoma libraries have been screened alongside their pre-fusion splenocytes. In these cases, often far fewer cells (as compared with chicken spleen) are used in each GEM prep, and fewer positive events are seen.

For the integrity of the assay, it is essential that any antibody profile in a GEM is derived from the secreted output of a single cell. Monoclonality is maintained statistically by generating GEM preps wherein the GEMs outnumber the antibody-secreting cells by a 5:1 or greater ratio. Conversely, the number of reporters (beads or cells) outnumbers the GEMs by a 5:1 or greater ratio to ensure that every GEM contains multiple reporters. There are more total cells than GEMs in a preparation, but the other cells are not B cells or are non-secreting B cells and therefore do not interfere with the assay. With respect to amplification of antibody genes, the presence of bystander non-secreting B cells is a theoretical concern and may contribute to ‘wrong’ sequences being amplified. However, the preponderance of mRNA is biased in favor of the highly secreting clone, and in practice we have not seen an impact on antigen-specific antibody recovery that could be attributed to cell loading densities. We have attempted to enrich the secreting B-cell population by magnetic separation or other methods, including isolation of splenic germinal centers, but these approaches have never proven worthwhile because of cellular losses and a reduction in cellular viability. Our strategy focuses on getting target cells out of the animal, through the screening process, and converting their mRNA into amplified DNA as quickly as possible.

For most projects, having two or three reporters in the GEM is sufficient to meet the design goals. Differentiation of beads can be done on the basis of color, as described in the examples above, but can also be done on the basis of size or morphology (Fig. 1d). With beads alone, 3 colors × 3 sizes can provide a 9-plex assay.

As shown in our DR4/DR5 example, cellular reporters can be used to screen directly for biological activity in GEMs. Not all cellular assays are adaptable, however. Due to the nature of our microscopy platform, a fluorescent output signal is required. Additionally, the signal needs to be relatively long-lived because the GEM incubation time is several hours to allow critical antibody concentration to be achieved. Thus, assays such as Ca^++^ flux cannot be incorporated into a GEM format, but assays in which cells harbor a reporter gene, such as GFP, which can be activated upon stimulation, can be useful in the GEM assay.

Extracting the critical information from each GEM requires a microscopy environment, because the content is contained in the spatial relationship between fluorescent signals within the GEM and the morphology of the reporters generating those signals. The key metric involves colocalization of fluorescent signals: one color is reserved to visualize the antibody and the other colors and morphologies are used to identify the reporters. This type of analysis is not possible in a classical flow cytometry-based system because simple quantitation of fluorescence is not sufficient to decode the GEM, regardless of the speed at which the system may operate.

It has been suggested that automated imaging and picking would improve GEM screening throughput, but in fact this would actually slow down the process. Capturing all the spatial information in a GEM would require imaging at relatively high magnification with multiple filters and could take up to 3 min to acquire a 3D-stack for each GEM. The longer it takes to decode and pick GEMs the lower the viability of the B cells, resulting in poor recovery at the RT-PCR stage. Therefore, we have opted for live visualization, using a microscope equipped with a triple filter that allows all colors in the GEM to be seen simultaneously. In this context, colocalization can be seen as a ‘hue shift’ producing, for example, the appearance of a yellow bead when a green bead is illuminated with a red secondary antibody. These colocalization events coupled with other visual cues about the reporter, such as morphology (e.g. ‘bead or cell’ or ‘big bead or little bead’), allows the operator to make a quick decision to collect the GEM.

## Concluding remarks

Within the biotechnology community, manual microscopy and manual clone picking are almost reflexively associated with low throughput. However, a single operator using the GEM assay can pick 200 or more candidates within a few hours in a single experiment. These candidates are the ‘best’, as defined by their specificity, biological activity, species cross-reaction or other parameters built into the GEM assay, from a preparation containing 5–100 million B cells. In a few hours, the GEM assay eliminates six logs of off-target material before molecular cloning is initiated. In a typical campaign, one 96-well plate of GEMs yields 20–30 unique sequence clones that meet the performance profile sought in the GEMs. For larger drug discovery campaigns, we typically immunize several birds and make multiple GEM preparations per bird from which we quickly and cost effectively generate several hundred antibody candidates. In contrast, microfluidic and robotic platforms typically have batch sizes of <1 million cells [56,57] and require a large investment in infrastructure.

The general philosophy driving development of the GEM assay has been identification of rare antibodies using color, morphology and orthogonal comparisons of reporters before initiating the time consuming molecular biology needed for clonal isolation and antibody expression/confirmation. The presumption of rarity for the most desirable clones precludes a deep sequencing approach, which, in stark contrast, biases toward the most prevalent sequences. A direct comparison of these approaches has yet to be made, but the stochastic process through which antibodies are created *in vivo* suggests that rare antibodies will be most effectively identified by technologies aimed at the tails rather than the mean of a normal distribution.

We consider the screening of avian B cells, a powerful application of the GEM technology for drug discovery; however, chicken antibodies such as those described in this report would require humanization prior to clinical development. A number of effective approaches have been described for humanizing chicken antibodies on a molecule by molecule basis [58–60]. In addition, there have been recent advances in the genetic engineering of the chicken immunoglobulin loci [61–63], including the completion of a transgenic chicken that produces human sequence antibodies. The antibody repertoire of such animals is expected to be a rich source of drug candidates for a variety of therapeutic targets.

## Funding

Funding to pay the Open Access publication charges for the article was provided by Crystal Bioscience, Inc.
